# Diagnostic significance of serum lncRNA HOTAIR and its predictive value for the development of chronic complications in patients with type 2 diabetes mellitus

**DOI:** 10.1186/s13098-021-00719-3

**Published:** 2021-09-08

**Authors:** Huiyun Wang, Yu Xia, Yanxia Zhang

**Affiliations:** Department of Health Comprehensive Geriatrics, Yidu Central Hospital of Weifang, No. 4138, Linglongshan Road, Weifang, 262500 Shandong China

**Keywords:** diagnosis, prognosis, HOTAIR, T2DM

## Abstract

**Background:**

Type 2 diabetes mellitus (T2DM) affects the social economy and quality of life, and has become a major threat to human health. This observation aimed to study the possibility of serum HOTAIR as a diagnostic index in patients with T2DM and to explore the prognostic potential of HOTAIR in the development of T2DM.

**Methods:**

The expression of HOTAIR in serum of 96 patients with T2DM and 82 healthy controls was detected by the qRT-PCR technique. The related biochemical indexes of all participants were determined, such as total cholesterol (TC) and fasting blood glucose (FBG). The value of serum HOTAIR in the diagnosis of T2DM in the two groups was analyzed by the ROC curve. Moreover, the prognostic value of HOTAIR on T2DM was examined by the K-M curve and COX multivariate analysis.

**Results:**

The results of the qRT-PCR analysis showed that the serum level of HOTAIR in patients with T2DM was significantly higher than that in healthy controls. ROC analysis showed that HOTAIR in serum was a diagnostic factor of T2DM. Further multivariate analysis showed that HOTAIR could be an independent biomarker in the prediction of chronic complications for T2DM patients, such as diabetic retinopathy and diabetic nephropathy.

**Conclusions:**

We found the augment of HOTAIR expression was a character of T2DM. The high expression of serum HOTAIR was a potential non-invasive diagnostic marker and independent prognostic factor in patients with T2DM.

## Background

Type 2 diabetes mellitus (T2DM) is a common metabolic disorder in the clinic, characterized by high blood glucose and complex pathogenesis, including insulin-resistant β cell failure [1, 2]. The etiopathogenesis of T2DM mainly involves insulin resistance and insulin secretion deficiency [3]. In addition, genetic, environmental, and glycolipid dysfunction also play an important role in the occurrence and development of diabetes, which are the main risk factors for vascular lesions [4, 5]. The vast majority of diabetic complications, if not treated in time, will not only reduce the quality of life of patients but may be life-threatening [6, 7]. Early prediction and effective treatment of diabetic complications are currently the focus of research [8]. With the development of molecular biology, it is necessary to explore the pathogenesis of diabetes on the molecular level and find the related prediction factors.

Long non-coding RNA (lncRNA) is a class of RNA longer than 200 nucleotides [9]. A variety of lncRNAs participate in the occurrence and development of T2DM, which was reflected by more and more shreds of evidence. A study finds that lncRNA ENST00000588707.1 is related to glucose metabolism and serves as a feasible biomarker in the field of T2DM [10]. A next-generation sequencing about T2DM discovers that the expression of MALAT1 and MIAT is raised and these abnormal levels have associations with insulin resistance [11]. LncRNA NONRATT021972 is at an increased level in patients with T2DM and its interference may release neuropathic pain and inflammation [12].

In recent years, the investigation has found that HOTAIR is abnormally expressed in several disorders involving T2DM and acts as a satisfactory predictor. The human lncRNA HOTAIR (HOX transcript antisense intergenic RNA) is a 2364 bp lncRNA transcribed from a 6449 bp gene [13]. HOTAIR locates on chr.12q13.13 and consists of six exons [14]. The half-life of HOTAIR has cell-specific variations [15]. The HOTAIR transcript’s half-life is approximately 4 h in HeLa cells [16]. Shaker et al. [17] provide the expression of HOTAIR is raised in diabetic retinopathy patients and it may distinguish diabetic retinopathy from nondiabetic retinopathy. Besides, a report manifests that hyperglycemia contributes to the enhanced HOTAIR expression in HRECs [15]. In diabetic cardiomyopathy, HOTAIR participates in the development of Akt phosphorylation and viability of cardiomyocytes [18]. Additionally, in an experiment about the function of HOTAIR on mice treated with high-fat food, the expression of HOTAIR is significantly elevated, which documents a close correlation between HOTAIR and T2DM [19]. Nevertheless, the clinical significance of HOTAIR in T2DM is not clear.

Hence, this study included T2DM patients and healthy people to detect the change of HOTAIR expression. Moreover, the diagnostic and prognostic values of HOTAIR were identified in our investigation.

## Materials and methods

### Patients and sample collection

Ninety six newly detected T2DM patients in Yidu Central Hospital of Weifang were selected and divided into the T2DM group. A total of 82 healthy subjects who received physical examinations in the same period were selected and set as the healthy control group. The inclusion criteria of the T2DM group were in accordance with the criteria for diagnosis and classification of diabetes recommended by the World Health Organization in 1999, namely fasting venous plasma glucose ≥ 7.0 mmol/L [20]. All participants had not received any antidiabetic drugs or insulin treatment before. Patients with other types of diabetes, severe cardiovascular diseases, and malignant tumors were excluded. The elbow venous blood of the two groups was collected in the morning and centrifuged with 1500 r/min at room temperature for 5 min. The serum samples were collected and stored at − 80 ℃. No blood was presented in the serum samples. This study was approved by the Ethics committee of Yidu Central Hospital of Weifang.

All participants were followed up for 5 years after being included in this study. Some chronic complications, including microvascular complications and macrovascular complications, were recorded. Microvascular complications included diabetic nephropathy, diabetic neuropathy, and diabetic retinopathy; macrovascular complications included cerebrovascular disease, cardiovascular diseases, and so on. The follow-up was completed if the endpoint event occurred or a 5-year assessment was conducted.

### Detection of HOTAIR expression in T2DM patients

The total RNA of serum was extracted by the TRIzol method according to the instructions of the TRIzol LS kit. In short, 1 ml TRIzol was mixed with per 200 µl serum sample for 5 minutes, and then 200 µl chloroform was added to the previous mixture. The supernate was obtained after being centrifuged and isopropanol was added to precipitate RNA. After that, RNA was washed with 75 % ethanol and solubilized with RNase-free water. The OD260/280 value was detected by NanoDrop™ spectrophotometer and only samples with OD260/280 ratio of 1.8–2.0 could be used for cDNA synthesis. Then, the concentration of total RNA was measured with Qubit 3.0. The 5 × gDNA digester Mix in the reverse transcription kit (Yeason, China) was used to remove the residual genomic DNA. A total of 1 µg extracted total RNA was reverse transcribed according to the instructions of the reverse transcription kit (Yeason, China, 11121ES60) to synthesize cDNA. PCR reaction was carried out according to the instructions of the TB Green^®^ Premix Ex Taq™ II (Takara, Japan, RR820A). Namely, 10 µl TB Green Premix Ex Taq II, 0.4 µl ROX Reference Dye II (50X), 0.8 µl Forward Primer (10 µM), 0.8 µl Reverse Primer (10 µM), 6 µl RNase-free water, and 2 µl cDNA were mixed on ice. The primers were as follows: HOTAIR forward, 5’-GCAGTGGAATGGAACGGATT-3’, HOTAIR reverse, 5’-CGTGGCATTTCTGGTCTTGTA-3’; β-actin forward, 5’-CCACCATGTACCCAGGCATT-3’, β-actin reverse, 5’-CGGACTCATCGTACTCCTGC-3’. The expression level of HOTAIR in each group of samples was calculated using the 2-DeltaDeltaCt method. β-actin was used as the internal control [21, 22].

### Statistical analysis

GraphPad Prism and SPSS software were used for statistical analysis. The measurement data of normal distribution were expressed by mean ± standard deviation (x ± s). The Kolmogorov-Smirnov test was used to detect whether data conformed to normal distribution. Continuous variables between the two groups were compared by the independent samples t test. Non-parametric data were compared with the Mann-Whitney U test, and categorical data were compared with the chi-square test. Clinical significance of HOTAIR was elucidated by ROC curve and K-M curve. COX multivariate analysis was used to verify whether HOTAIR was an independent indicator in T2DM. *P* < 0.05 is statistically significant.

The estimation of sample size was based on preliminary data regarding a study power of 80 % (alpha = 0.05, beta = 0.2) and an effect size of 20%. The sample size required for each group was calculated as 70 and the total sample size required was 140.

## Results

### Baseline clinicopathological features of all participants

There were 44 males and 38 females in the control cohort with an average age of 51.07 ± 6.38 years, and 51 males and 45 females in T2DM group with a mean age of 50.95 ± 6.45. There were no obvious differences in gender, age, and BMI between healthy people and the T2DM group (*P* > 0.05, Table 1). The alternation of LDL, TC, TG, and hypertension in the T2DM group was not statistically significant (*P* > 0.05, Table 1). The concentration of HDL was lessened in the T2DM patients (*P* < 0.01, Table 1). The levels of FBG in the T2DM group were significantly higher than those in the healthy control group (*P* < 0.001, Table 1). Besides, the HbA1c content was elevated in the T2DM group compared with the control group (P < 0.001, Table 1).

**Table 1:** Clinical data of the study population

Variables	All subjects (n = 178)		*P* value
	Healthy individuals (n = 82)	T2DM patients (n = 96)	
Gender (male/female)	44/38	51/45	0.943
Age (years)	51.07 ± 6.38	50.95 ± 6.45	0.824
BMI (kg/m^2^)	23.91 ± 2.09	24.26 ± 2.05	0.489
HDL (mmol/L)	1.36 ± 0.15	1.31 ± 0.36	< 0.01
LDL (mmol/L)	2.42 ± 0.37	2.43 ± 0.48	0.671
TC (mmol/L)	4.59 ± 1.18	4.84 ± 0.96	0.122
TG (mmol/L)	1.07 ± 0.35	1.05 ± 0.35	0.736
FBG (mmol/L)	5.04 ± 0.58	7.77 ± 0.44	< 0.001
HbA1c (%)	4.66 ± 1.01	6.59 ± 1.53	< 0.001
Hypertension (yes/no)	38/44	46/50	0.834

The data of age, BMI, HDL, LDL, TG, FBG were analyzed by Mann-Whitney U-test. The differences in TC and HbA1c were analyzed by the t test method. The chi-square test was used to calculate the differences in gender and hypertension

Data are expressed as n or mean ± standard deviation

*BMI* body mass index; *HDL* high-density lipoprotein; *LDL* low density lipoprotein; *TC* total cholesterol; *TG* triglycerides; *FBG* fasting blood glucose

### HOTAIR expression in T2DM

**Fig. 1:**
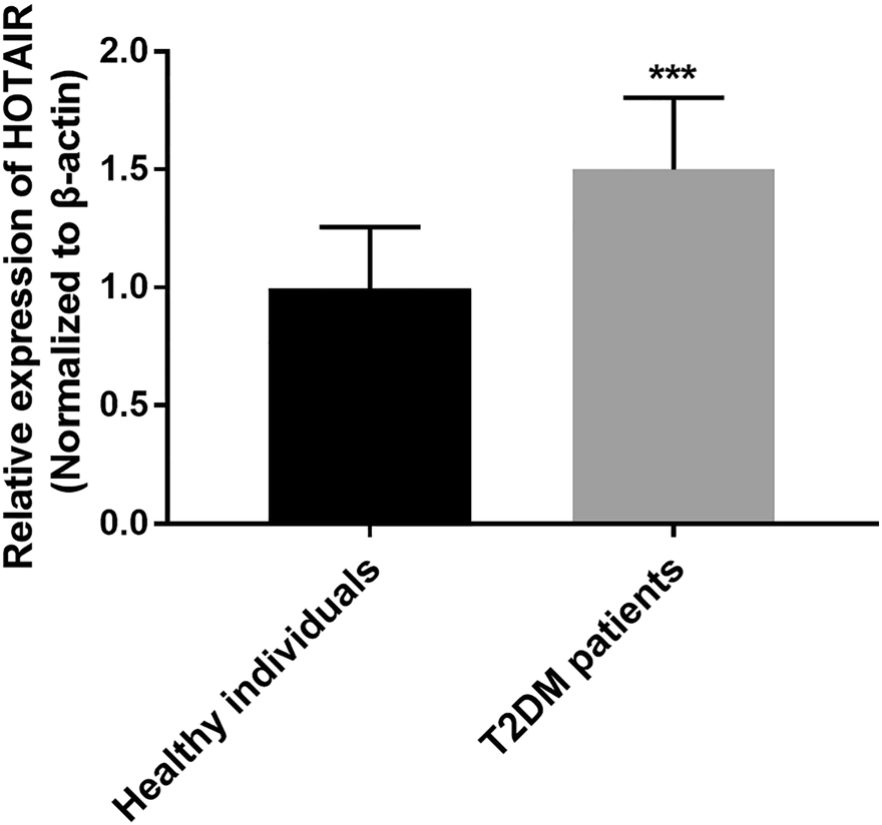
Elevated expression of HOTAIR was found in 96 patients with T2DM compared with 82 healthy individuals. The method of calculating relative HOTAIR expression was the Livak and Schmittgen method, and the t test was used to compare the differences. ***P < 0.001

The expression of HOTAIR in T2DM patients and healthy individuals was identified. As demonstrated in Fig. 1, the expression of HOTAIR was raised in the T2DM patients in comparison with control individuals, insisting T2DM might contribute to the enhancement of HOTAIR expression (*P* < 0.001). The expression of HOTAIR in T2DM patients was 1.509 times that of healthy individuals.

### Diagnostic performance of HOTAIR

**Fig. 2:**
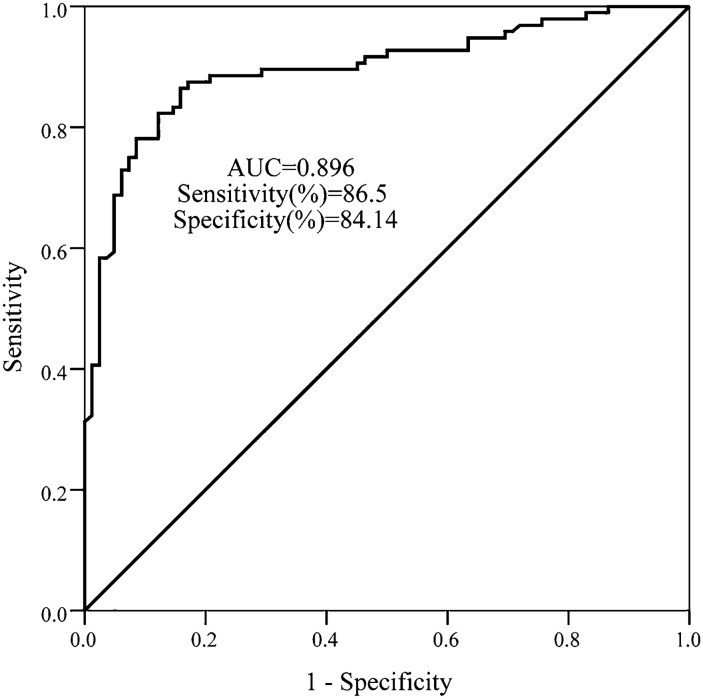
The diagnostic value of HOTAIR for T2DM patients. A total of 96 T2DM patients and 82 healthy controls were included and a ROC curve was applied to analyze the diagnostic value

Considering the alternation of HOTAIR expression in T2DM patients, the diagnostic possibility of HOTAIR was examined by the ROC curve. The AUC of HOTAIR was 0.896 (standard error, SE = 0.024), and the percentage of sensitivity and specificity was 86.50% and 84.14 % respectively at the cut-off value of 1.216 (Fig. 2). This finding substantiated HOTAIR could discriminate T2DM patients from control individuals.

### Prognosis of HOTAIR in T2DM

**Fig. 3:**
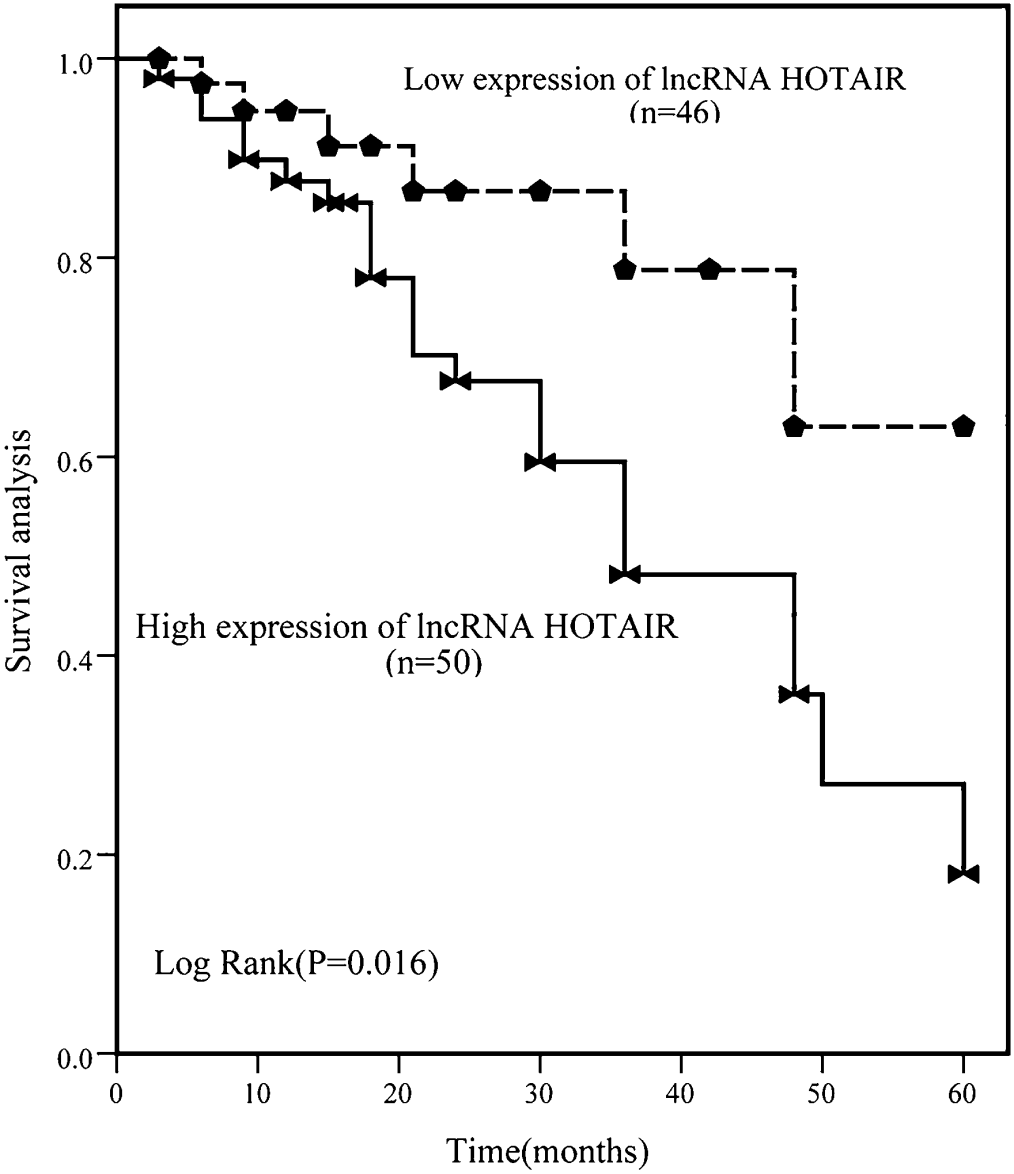
Diagnostic significance of HOTAIR in T2DM complications was analyzed by K-M curve. There were 46 patients in the low HOTAIR expression group and 50 patients in the high HOTAIR expression group

Based on the average expression of HOTAIR in T2DM patients, all patients were divided into the low HOTAIR expression group and high HOTAIR expression group. Fifty patients were included in the high expression of HOTAIR group and others were composed of the low expression group. The follow-up revealed that 35 patients had T2DM complications, including 23 patients with microvascular complications, 16 patients with trace urine protein, and 4 patients with retinopathy. The analysis based on the chronic vascular diseases showed that individuals with high HOTAIR expression were more likely to develop chronic complications (*P* = 0.016, Fig. 3).

When the healthy control population was used as the dichotomous variable, the results of multivariate regression analysis showed that HOTAIR was an independent risk factor for T2DM complications (HR = 3.577, 95 % CI 1.349–9.487, *P* = 0.010, Table 2). Further, age, sex, BMI, HDL, LDL, TC, TG, and hypertension were used for correction (*P* > 0.05). FBG was an independent risk factor for T2DM complications (HR = 2.160, 95% CI 1.017–4.586, *P* = 0.045, Table 2). The level of HbA1c was an independent biomarker of T2DM (HR = 2.484, 95 % CI 1.076–5.737, *P* = 0.033, Table 2).

**Table 2:** Multivariate Cox analysis of clinical characteristics in relation to overall survival

Factors	Multivariate analysis		
	HR	95 % CI	*P*
LncRNA HOTAIR	3.577	1.349–9.487	0.010
Age	0.763	0.350–1.663	0.497
Gender (male/female)	0.815	0.374–1.772	0.605
BMI (kg/m^2^)	0.742	0.325–1.695	0.479
HDL (mmol/L)	0.492	0.229–1.055	0.069
LDL (mmol/L)	1.168	0.551–2.473	0.685
TC (mmol/L)	1.479	0.665–3.285	0.337
TG (mmol/L)	0.632	0.299–1.334	0.229
FBG (mmol/L)	2.160	1.017–4.586	0.045
HbA1c (%)	2.484	1.076–5.737	0.033
Hypertension	0.707	0.338–1.480	0.357

*BMI* body mass index; *HDL*high-density lipoprotein; *LDL*low density lipoprotein; *TC* total cholesterol; *TG*triglycerides; *FBG* fasting blood glucose

## Discussion

T2DM is a common type of diabetes, which basically has a high incidence in the elderly population [23]. T2DM is caused by environmental influence, genetic factors, or metabolic dysfunction [24]. T2DM mainly leads to insulin dysfunction and endocrine function disorders, which will also lead to adipocyte lesions in patients [25]. The high morbidity and mortality of T2DM seriously affected socio-economic condition, life quality, and human health [26]. Chronic vascular complications are the leading causes of disability and death in patients with diabetes [27]. Most T2DM patients died of macroangiopathy, and microangiopathy is also the main cause of blindness and renal failure in T2DM [28]. Therefore, chronic vascular complications of diabetes have received more attention. However, the etiology and pathogenesis of T2DM have not yet been fully elucidated.

A large number of recent studies have shown that lncRNAs participate in the occurrence and development of diabetes and play key regulatory roles. In an experiment, lncRNA MALAT1 is at a raised level in mice with insulin resistance and may be a target of T2DM therapy [29]. MEG3 is elevated in patients with T2DM and its levels are in direct proportion to the concentration of glucose, underlying a novel perspective of pathogenesis of T2DM [21]. Research articles on the significance of HOTAIR have been widely published in the literature [30]. In a report about the function of HOTAIR, HOTAIR facilitates the progress of glucose uptake in the LPS-triggered macrophages via modulating upstream genes of glucose metabolism [31]. Another study unveils that the highly expressed HOTAIR can regulate glucose levels by promoting insulin transcription-related genes, which provides the expression of HOTAIR that may be closely relative to glucose metabolism [32]. Besides, in pancreatic adenocarcinoma, HOTAIR is highly expressed in tumor tissues and can promote energy metabolism of cancer cells by enhancing glucose uptake, lactate production, and ATP synthesis [33]. However, research on HOTAIR in the field of the pathogenesis of T2DM is scarce and its expression in peripheral blood of T2DM patients has not been elucidated yet. In this investigation, the expression of HOTAIR was elevated in T2DM patients, providing that HOTAIR might be involved in the development of T2DM. A report published in 2018 also finds that the expression of HOTAIR is elevated in T2DM patients, which further verified our results [11].

The clinical values of lncRNAs on T2DM have been discussed by several researchers. In a pooled research, lncRNAs are likely to be predictive indicators for T2DM patients after analyzing 7 previous publications [34, 35]. In particular, little is known about the significance of HOTAIR in the prognosis and diagnosis of T2DM. The diagnosis of HOTAIR for T2DM patients was proved in this study, which was derived from the finding that HOTAIR might distinguish T2DM patients from healthy persons with high specificity and sensitivity. LncRNAs have also been approved as prognostic markers for many diabetic complications. In research about diabetic retinopathy, the expression of HOTAIR is raised in patients and used as a novel biomarker in diabetic retinopathy [17]. Besides, the AUC of 0.878 reasons that serum HOTAIR may distinguish diabetic cardiomyopathy patients from healthy individuals [36]. In the current study, the prognostic value of HOTAIR on the diabetes-relative complications was detected by the K-M curve. This analysis indicated that abundantly expressed HOTAIR might be an indicator to predict the occurrence of chronic complications. Moreover, further analysis provided that HOTAIR might be an independent biomarker in the prognosis of T2DM. These findings provided a viewpoint that HOTAIR might associate with the pathogenesis of T2DM and can be used as a significant biomarker. However, some limitations existed in this study, including a small sample size, possible selection bias of patients, and insufficient clinical data. The absence of data on smoking and uric acid from all individuals, which might limit this investigation.

Collectively, this study validated T2DM contributed to the increased HOTAIR levels. The abundance of HOTAIR indicated an increased probability of suffering T2DM. High expression of HOTAIR might be a prognostic indicator because it could lead to a worse outcome of chronic complications. Besides, HOTAIR and FBG were independent biomarkers of T2DM respectively.

## Data Availability

The datasets used and/or analyzed during the current study are available from the corresponding author on reasonable request.

## Abbreviations

T2DM: Type 2 diabetes mellitus
BMI: Body mass index
HDL: High-density lipoprotein
LDL: Low density lipoprotein
TC: Total cholesterol
TG: Triglycerides
FBG: Fasting blood glucose
